# Predicting the clinical performance of dental students with a manual dexterity test

**DOI:** 10.1371/journal.pone.0193980

**Published:** 2018-03-08

**Authors:** Diva Lugassy, Yafi Levanon, Raphael Pilo, Asaf Shelly, Gal Rosen, Avi Meirowitz, Tamar Brosh

**Affiliations:** 1 Department of Oral Rehabilitation, The Maurice and Gabriela Goldschleger School of Dental Medicine, Sackler Faculty of Medicine, Tel Aviv University, Tel Aviv, Israel; 2 Department of Occupational Therapy, The Stanley Steyer School of Health Professions, Sackler Faculty of Medicine, Tel Aviv University, Tel Aviv, Israel; 3 Private clinic, Rishon Le’Tzion, Israel; 4 Department of Oral Biology, The Maurice and Gabriela Goldschleger School of Dental Medicine, Sackler Faculty of Medicine, Tel Aviv University, Tel Aviv, Israel; IUMPA - Universitat Politecnica de Valencia, SPAIN

## Abstract

Dentists must be skilled when using dental mirrors. Working with mirrors requires spatial perception, bimanual coordination, perceptual learning and fine motor skills. Many studies have attempted to determine the predictors of manual skills among pre-clinical students, but consensus has yet to be reached. We hypothesized that valid and reliable occupational therapy test performance regarding indirect vision would differ between dental students and junior dentists and would explain the variance in manual skill performance in pre-clinical courses. To test this hypothesis, we applied the Purdue Pegboard test and O’Connor Tweezer Dexterity test under different conditions of direct and indirect vision. We administered these tests to students in phantom-head academic courses in 2015 and 2016 and to junior dentists. Students performed the tests at three time points: before phantom training (T0), at the end of the training (T1) and in the middle of the following year of study (T2). Dentists performed the same tests twice at 1^st^ and 2^nd^ trials one week apart. The results showed that indirect tasks were significantly more difficult to perform for both groups. These dexterity tests were sensitive enough to detect students’ improvement after phantom training. The dentists’ performances were significantly better than those of students at T0, specifically with regard to the use of tweezers under direct and indirect vision (the O’Connor test). A regression analysis showed that students’ manual grades obtained at the beginning of the phantom course, their performance on the Purdue test using both hands, and their performance on the O’Connor test under indirect vision predicted phantom course success in 80% of cases. The O’Connor test under indirect vision is the most informative means of monitoring and predicting the manual skills required in the pre-clinical year of dentistry studies.

## Introduction

Dentistry is a medical profession that requires fine motor skills [1], hand-eye coordination [2] and spatial perception [3]. Furthermore, perceptual learning is needed for indirect visualization through a mirror [4–6]. However, admission to dental schools worldwide is often based on academic and cognitive factors [7]. Studies have shown that the correlation between the high school grade point average (GPA) of dentistry candidates and their performance in pre-clinical practical courses is poor [8,9]. Thus, a large percentage of students are unable to successfully fulfill the requirements of pre-clinical courses [10], and graduation is difficult [11]. Many studies have attempted to identify a screening tool that can more precisely predict the future performances of students in pre-clinical practical courses [12].

Manual dexterity tests are ordinarily used in the field of occupational therapy to diagnose fine motor disorders of injured patients as well as the adaptability of healthy individuals to specific fine assembly jobs. Over the past 80 years, various studies have been conducted using existing manual dexterity tests from occupational therapy to examine the association between fine motor skills and the performance required to pass pre-clinical dentistry courses. These tests and measures include block carving [13], the tremometer test [14], the two-hand coordination machine [14], the O’Connor Tweezer Dexterity Test [15] and the Purdue Pegboard Test [16], among others [17,18]. However, no consensus has been reached regarding the best predictive test [19]. For example, Lundergan et al. [15] concluded that the O’Connor test has low predictive ability, whereas de Andrés et al. [20] reported that this test predicts poor student performance and identifies low-performing students in advance. Another study found that a stainless-steel frame simulating the mouth and containing two plastic arches with 32 holes was more comparable to dental tasks and demonstrated that this test could be used as an additional screening tool for dental students [21]. The University of Hamburg, Germany demonstrated that the wire-bending test is an additional and valuable screening tool for dental school applicants [9].

Dentistry requires individuals to work simultaneously with both hands using a dental mirror to perform tasks. Previous studies have investigated the indirect vision skills of students using perceptual tests from psychology as potential preadmission predictors [4–6]. Disagreements have been reported regarding the conclusions of these studies. Although indirect vision skills measured by psychology tests have been studied, few dexterity tests from the occupational therapy field that measure fine motor skills have been conducted under indirect vision conditions.

In this study, we used a new approach by applying the Purdue Pegboard test and the O’Connor Tweezer Dexterity test under different conditions of direct and indirect vision. An indirect approach might be more suitable to assess dental performance because it measures not only fine motor skills but also perceptual learning and spatial perception. We hypothesized that valid and reliable occupational therapy test performance under indirect vision would differ between dental students and junior dentists and would explain the variance in manual skill performances among dental students. Therefore, the aims of the study were as follows: 1. to evaluate manual skills through a new approach using dexterity tests under conditions of direct and indirect vision among dental students and dentists; 2. to determine whether these tests are sensitive to the improvement of students’ manual skills after training in the pre-clinical course and whether they reflect the development of these skills over time; 3. to evaluate the differences in tests performance between dental students and dentists; 4. determine which of the tests are capable of predicting the success of dental students in the pre-clinical year.

## Materials and methods

### Participants

A total of 95 participants were included in this study, comprising students and junior dentists. The students (N = 65) were in their first year of practicing on a phantom head. The dentists (N = 30) were junior dental practitioners with three to five years of clinical experience who worked in public health clinics. For anonymity, each participant was randomly assigned an identifying number known only to the researcher. We initiated the study by testing 39 students (24 females, 15 males, age 25.8±3.33 years, range 23–40 years) in the 2015 academic year (cohort 1). Cohort 1 students constituted the entire student body that year; thus, we avoided selection bias. The experiment was repeated over the next academic year (2016) with 26 students (18 females, 8 males, age 24.6±1.49 years, range 23–28 years, i.e., cohort 2) to validate the results obtained for 2015. However, only half of the 2016 students agreed to participate in this research. Selection bias was controlled by comparing the clinical grades of students who participated in the study to those who did not participate in the study from the same class. In addition, 30 dentists (12 females, 18 males, age 30±2.96 years, range 26–38 years) volunteered for this study in 2015. All participants signed an informed consent document approved by the ethics committee of Tel Aviv University.

### Dexterity tests

#### Purdue Pegboard test

This test is composed of a board with two columns of holes. The distal portion of the board includes 4 cups: one right and one left cup containing 25 pins each, a cup containing washers and a cup containing collars. The test includes four tasks. As many pins as possible must be inserted into holes on the board in 30 s for the first three tasks and in 60 s for the last task. For each of the four tasks, the number of inserted pins represents the score. The tasks differ in the following ways:

1. Purdue direct using dominant hand (PD-DH).
2. Purdue direct using non-dominant hand (PD-NDH).
3. Purdue direct using both hands (PD-BH). This task is accomplished by holding one pin with each hand and inserting them into two holes simultaneously.
4. Purdue direct assembly task (PD-A). During assembly, the participant works continuously with both hands to construct four parts starting with a pin, threading a washer and a collar, and ending with an additional washer.

Following the four tasks in direct vision, the participants performed the same tasks in indirect vision (see below for the procedure) as follows: PIND-DH, PIND-NDH, PIND-BH, PIND-A.

#### O’Connor Tweezer Dexterity test

This test consists of a board that has 100 holes and a cup that holds 100 pins. The participant inserts all 100 pins using tweezers with the dominant hand. This test was performed under conditions of direct and indirect vision: O’Connor Tweezer Direct (O-D) and O’Connor Tweezer Indirect (O-IND).

The time needed to insert all the pins into the holes was considered the score.

### Student study

**Fig 1:**
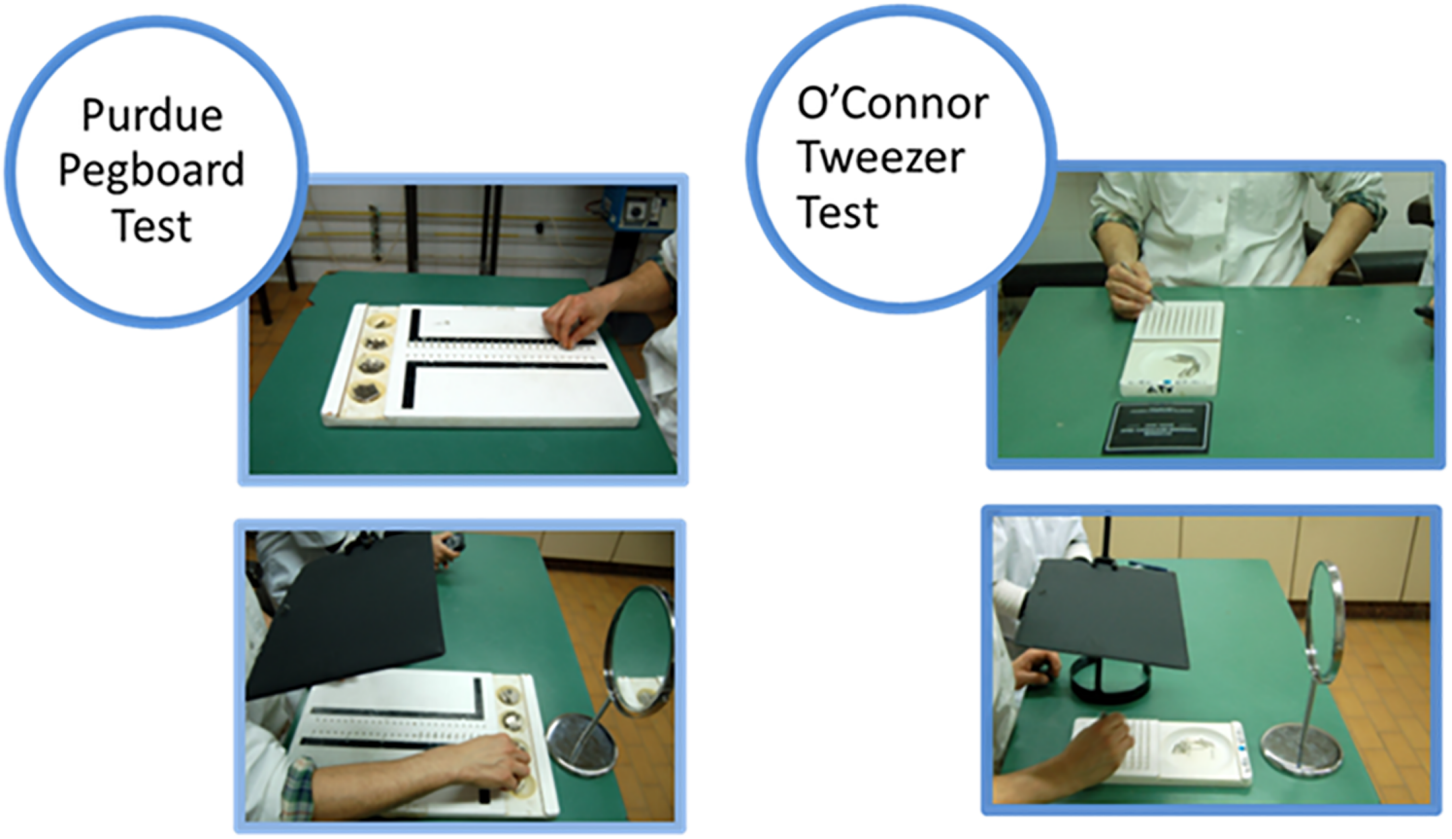
Purdue Pegboard test and O’Connor Tweezer test under direct (Top) and indirect (Bottom) vision.

Both dexterity tests were conducted twice: initially with direct vision and then with the novel indirect vision using a mirror. The indirect vision is accomplished by hiding the test’s board with a blackboard shield to prevent a direct view on the board and to force the participants to observe the tasks through a mirror (Fig 1). All students were tested in a quiet, comfortable, well-lit room to avoid distractions. Before performing each stage, a single instructor read identical and clear instructions to each participant from written notes. Between the tasks, the students were allowed to refresh themselves by walking around the room.

The experiments were performed at three time points for cohort 1 (T0, T1, and T2) but only at T0 and T1 for cohort 2:

T0: before phantom training (beginning of phantom course);
T1: after phantom training (end of the phantom course, 8 months after T0);
T2: in the middle of the following year (15 months after T0). This time point was included to verify the assimilation of dexterity over time, serving as a preservation test.

During each period, all students performed the same order of testing: four tasks of PD, four tasks of PIND, O-D, and O-IND. The time necessary to administer the battery of all tests during each period ranged from 60 to 90 minutes per participant.

Students’ dental performance was assessed at the beginning of the course via an initial phantom grade and at the end of the year via the final phantom grade. The initial phantom grade (Initial.Phantom) was the average grade routinely provided by the clinical instructors three weeks after beginning the phantom course based on preparing a Class 1 cavity and a Class 1 restoration in plastic phantom teeth. The final phantom grade (Final.Phantom) was the average grade of the three tasks routinely provided by the clinical instructors at the end of the operative dentistry course performed on plastic teeth. Three instructors provided the grades using several common criteria, such as outlines of the cavity preparation, cavity depth and other criteria. The grades are on a continuous scale of 0–100 whereas the passing grade is 60. Because the student identification numbers were coded, the instructors were unaware of the individual students’ results. The study was blinded in the sense that the principal investigator who conducted the dexterity tests was blind to the students’ final grades for the phantom course.

### Dentist study

Thirty experienced general practitioner dentists participated in this study. The inclusion criterion was three to five years of clinical experience. At 1^st^ trial, half of the dentists performed the Purdue tests and half performed the O’Connor tests under conditions of direct and indirect vision. At the 2^nd^ trial, eight dentists repeated the four PD and PIND tasks, and another eight repeated the O-D and O-IND tasks one week later. Repeated measurements were performed to examine test-retest reliability.

### Statistics

Pearson’s correlation coefficients were calculated: between the two manual dexterity tests and between tasks at T0 and T1 for students, between the students’ grades for the phantom course and their dexterity test scores (i.e., the explanatory variables). Test-retest reliability for dentists was analyzed via the standard error of measurement (SEM) [22] and Pearson’s correlation coefficient. Two linear regression models, the first one with all independent variables using the enter method, followed by a second one using the stepwise method with only three independent variables, as well as a logistic regression using the enter method were conducted to examine the strength and combination of the explanatory variables of the Final.Phantom. Comparisons between the dexterity test scores with direct and indirect vision were performed using paired t-tests at different times. To control the problem of alfa inflation, it was divided by the number of relevant comparisons. A between-group comparison of students and dentists was performed using an independent-samples t-test. All analyses were performed using SPSS version 20. Significant statistical differences were defined as p<0.05.

## Results

The correlations analyses between the two manual dexterity tests and between tasks at T0 and T1 for both cohorts of students (N = 65) were calculated (see S1 Appendix). Generally, moderate to high correlations among the two tests and between the tasks at T0 (-0.421<r<0.657, p<0.05) and T1 (-0.455<r<0.720, p<0.05) were found. Low to moderate correlations were found between the same tasks at TO and T1 (0.298<r<0.703, p<0.05). The test-retest reliability analyses of the dentists between the same tasks at 1^st^ trial and 2^nd^ trial showed high correlations (S2 Appendix).

**Fig 2:**
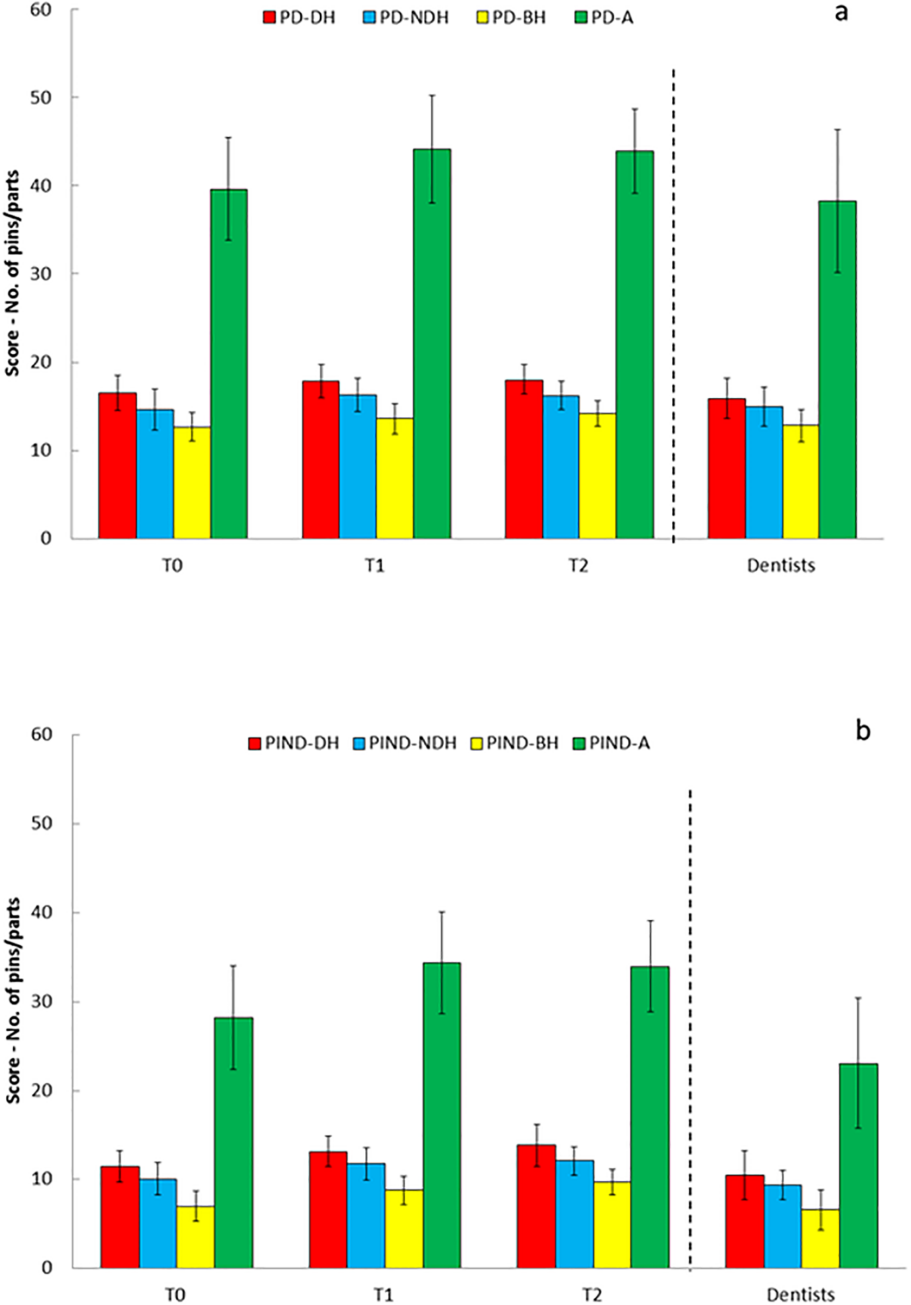
Mean and SD of the Purdue tests scores for cohort 1 (N = 39) and dentists (N = 15) under direct (a) and indirect vision (b).

Fig 2 (and S3 Appendix) presents the mean and standard deviation (SD) of the Purdue test scores under direct and indirect vision conditions across the three time periods for cohort 1 and the dentists at 1^st^ trial. The scores of both groups, students and dentists under direct vision were significantly higher than those under indirect vision (p<0.0005) across all times.

After practicing in the phantom lab, the scores of the students significantly increased for all tasks at T1 (p<0.0005). The scores were maintained for cohort 1 at T2 with no difference observed between T1 and T2 (p>0.05). No significant differences were found between the students at T0 and dentists at 1^st^ trial for all tasks performed under direct and indirect vision, with the exception of task 4 on the PIND-A (p<0.005), in which the students showed better performance. No significant differences (1 week apart, p>0.05) were found regarding the Purdue tests of the dentists (S4 Appendix).

**Fig 3:**
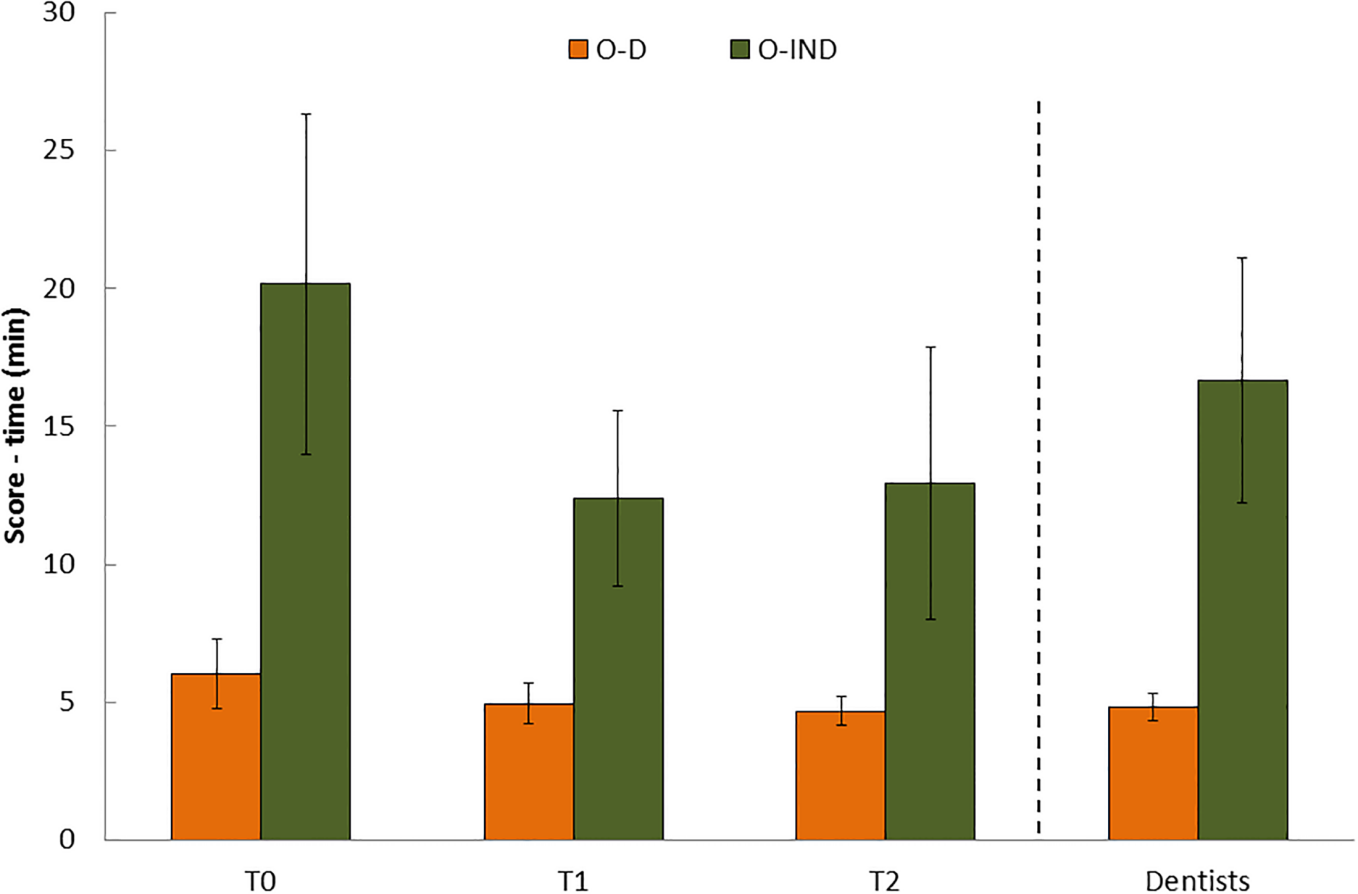
Mean and SD of the O’Connor Tweezer test scores for cohort 1 (N = 39) and the dentists (N = 15).

Similar trends were observed for the O’Connor test (Fig 3, and S3 Appendix): the scores under the indirect vision tasks were significantly higher than those on the direct vision tasks for all participants (p<0.0005) across all time periods. Note that the scores on the O’Connor test represent the time needed to insert 100 pins. Furthermore, significant improvements in task performance were observed between T0 and T1 for cohort 1 (p<0.0005). These improvements were pronounced with regard to the indirect task. No differences were found between T1 and T2 in O-D (p = 0.434) and O-IND (p = 0.939). Significant differences were observed between the students at T0 and dentists at 1^st^ trial for both the O-D and O-IND tasks (p<0.05). The dentists required approximately 15% less time to perform the tasks (S3 Appendix).

No significant differences were found between the 1^st^ trial and 2^nd^ trial (1 week apart, p = 0.07) of the O-D results of the dentists. However, significant differences were found for the O-IND (1 week apart, p = 0.015) in which 8% less time was needed to complete the task. The improvement of the students on the O-IND after 8 months of training (~35% at T1) was significantly higher (p<0.0005) than the improvement shown among the dentists at 2^nd^ trial after 1 week (~8%); that is, the percentage of improvement of the students was 4.5-fold higher than the dentists. The improvements of the dentists on the repeat test were attributed to learning memory, expressed as SEM = 4.28%. Student improvement on the O-IND test repeated after 8 months of practice (T1) was much greater. Therefore, this effect is not due to memory but is due to practice during the phantom course.

It was also noted that the largest coefficient of variation (CV) was found in the O’Connor test under indirect vision (15%-31%) compared to the direct vision (~11%-16%).

The Final.Phantom grades of cohort 1 were as follows: mean = 61.59, SD = 11.48, median = 64 and range scores = 30–76. To examine the strength of the association between the independent variables and the dependent variable (Final.Phantom), Pearson correlations were performed after examining the normal distribution of the variables (Table 1). Significant correlations were found with all dexterity tasks except for PD-DH and PIND-DH. The strongest associations with regard to the Final.Phantom grades concerned PD-BH, O-IND and Initial.Phantom. Furthermore, a linear regression analysis using the enter variable-selection method using all independent variables that revealed significant correlations in the previous Pearson correlation tests was applied. All independent variables explained 68% of the variance in the Final.Phantom grade (R^2^ = 0.684) and Adjusted R^2^ = 0.553 and produced a significant model (p<0.0005). This may be attributed to over-fitting of the independent variables. This analysis also showed that Initial.Phantom (β = 0.487), PD-BH (β = 0.446) and O-IND (β = -0.270) had the strongest influence on the Final.Phantom. Therefore, a linear regression analysis using the stepwise variable-selection method was performed using PD-BH, Initial.Phantom, and O-IND. This model explained 61.8% of the variance in the Final.Phantom grades (R^2^ = 0.618, p = 0.005). Initially, PD-BH was entered into the model and explained 37% of the existing association. Then, Initial.Phantom was added, and the explanatory variance increased to 56%. After adding O-IND to the model, the explained variance increased to 62% (Table 2).

**Table 1:** Correlations between student (Cohort 1, N = 39) independent variables at T0 and the Final.Phantom.

Independent variable	Correlation coefficient	P-value
PD-DH	0.185	0.288
PD-NDH	0.437**	0.009
PD-BH	0.607**	0.0005
PD-A	0.387*	0.022
PIND-DH	0.246	0.155
PIND-NDH	0.390*	0.021
PIND-BH	0.410*	0.014
PIND-A	0.447**	0.007
O-D	-0.399*	0.018
O-IND	-0.519**	0.001
Initial.Phantom	0.557**	0.001

*Correlation is significant at the 0.05 level.

**Correlation is significant at the 0.01 level.

**Table 2:** Linear regression analysis to predict the final grade in the phantom course using stepwise variable-selection method.

Independent variable	R	R^2^	Adjusted R^2^	R^2^ Change	Sig. F Change	β	P-value
Model 1	0.607	0.368	0.349	0.368	0.0005		
PD-BH						0.607	0.0005
Model 2	0.751	0.564	0.537	0.196	0.001		
PD-BH						0.515	0.0005
Initial.Phantom						0.452	0.001
Model 3	0.786	0.618	0.581	0.054	0.045		
PD-BH						0.419	0.002
Initial.Phantom						0.415	0.001
O-IND						-0.257	0.045

The students in cohort 1 were categorized based on their success or failure in the course (a passing grade was defined as more than 60 of 100 points). A logistic regression analysis was conducted using the three independent variables found in the previous model. The results were significant (p = 0.004) and led to correct predictions in 80% of the cases; in other words, 20% of the successes were erroneously classified as failures. The model with only PD-BH and O-IND also showed significant results (p = 0.017), making correct predictions in 74.3% of all cases.

The study was repeated with 26 students (half of the class) enrolled in the phantom course of the following year (cohort 2) (S5 Appendix). No significant differences were found between the two parts of the class regarding Initial.Phantom (p = 0.150) and Final.Phantom (p = 0.639).

No significant differences were found between the two cohorts regarding gender (p = 0.787), age (p = 0.075) and Final.Phantom grades (p = 0.206).

In the Purdue test, the scores under direct vision were significantly higher than those under indirect vision (p<0.0005) across the two time periods. After practicing in the phantom lab (T1), the scores significantly increased for all tasks (p<0.0005) except PD-DH (p = 0.07) and PIND-A (p = 0.188). Similar to cohort 1, no significant differences were found between the students and dentists at T0 for all tasks performed under direct and indirect vision except task 4 on the PIND-A (p = 0.0005).

Similar trends were observed for the O’Connor test (S5 Appendix): the scores under the indirect vision tasks were significantly higher than those of the direct vision tasks across two time periods (p<0.0005). Furthermore, significant improvements in task performance were observed between T0 and T1 (p<0.002). Significant differences were observed between the students at T0 and dentists at 1^st^ trial for the O-D (p<0.0005) and O-IND (p<0.0005) tasks. The student improvement on the O-IND after 8 months of training was significantly higher (p<0.001) than that shown among the dentists after 1 week. The PIND-BH (r = 0.412, p = 0.05) and O-IND (r = -0.417, p<0.05) tests indicated the strongest correlations with the final grades (for cohort 2: mean grades = 64.78, SD = 8.6, median = 68 and range = 43–75) whereas all other independent variables showed low non-significant correlations. No regression analysis was conducted on cohort 2 because of the small number of participants that also attributed to the statistical power of the correlations.

## Discussion

Our results show that performing the Purdue and O’Connor dexterity tests is appreciably more difficult under indirect vision. Both the students and the dentists had worse scores under this condition than under direct vision. Moreover, the differences observed between the direct and indirect vision conditions were much more pronounced for the O’Connor Tweezer Test than for the Purdue test (S3 Appendix). Among the students at T0, under indirect vision condition of the Purdue test resulted in scores that were approximately 34% lower (mean of the four tasks) than those obtained under direct vision, whereas on the O’Connor test, approximately 3.5-fold more time was required to complete the mission under indirect vision than under direct vision. Interestingly, significant differences were observed between the students at T0 and dentists at 1^st^ trial for the O-D and O-IND tasks: dentists performed 15% better than students did. As opposed to the O’Connor test, in the first three successive tasks of the Purdue tests, the students and dentists performed similarly. In the PIND-A, the students performed better. It is assumed that the Purdue test that involves the fingertips and lasts 30–60 s is less relevant to daily clinical practice than the O’Connor test, which involves the usage of tweezers and is much more time consuming. The PIND-A is the most complex task of the Purdue tests and requires cognitive and mental practice. It is postulated that during their studies, dental students are more involved with cognitive and mental practice than dentists and thus perform better that task [23]. The use of tweezers (O’Connor test) is more common in long-term dental procedures than working with fingers (Purdue test) on short-term tasks. Thus, the O’Connor test should predict the manual dexterity skills needed for clinical practice. After practicing during the phantom course (T1), the students’ performances improved, and they performed better than the dentists on the O-IND (Fig 3 and S3 Appendix). We conclude that working under indirect vision is a skill acquired during the pre-clinical year, a finding that is compatible with the results of other studies [5,6]. However, dentists continue to experience frustration in their attempts to use indirect vision throughout their careers, and they tend to avoid using dental mirrors for the maxillary arch [6].

Our hypothesis that occupational therapy tests under conditions of indirect vision would explain the variance in manual skills among dental students was also supported by the larger coefficient of variation (CV) under the indirect as compared to the direct tests, profoundly in the O’Connor test under indirect (31%). It can be assumed that tasks under indirect vision necessitate extra capabilities, such as spatial perception, orientation and perceptual learning in addition to fine motor skills, bimanual and hand-eye coordination.

Regression models enabled us to predict success in the phantom course (Table 2). Based on the performance of cohort 1, a logistic regression model predicted student success in the course with 74% accuracy by using performance on missions with both hands using the fingers for the Purdue test and using tweezers under indirect vision for the O’Connor test. These two tests could be used during the application process for dental students. Adding the initial phantom grade, which includes a cognitive understanding of the missions, academic knowledge and manual skills, increased the accuracy in predicting success in the phantom course by ~6%. Schmidt and Lee [24] claimed that if a test explains 70% or more of the variance in target skill performance, then the essential abilities that underlie this performance have likely been identified. Our study constructed a precise model that predicts student success in a phantom course.

Our hypothesis that occupational therapy tests, which are considered very reliable [15,25,26], would be sensitive to the improvement of students’ manual skills after training in the pre-clinical course was also supported. Higher scores were found at T1 versus T0. Positive transfer is defined as a situation in which practice on one motor task leads to significant improvement on the performance of another task. Researchers have debated whether positive transfer actually exists. Thorndike [27] and Osgood [28] concluded that this transfer depends on identical elements between two performances (i.e., for transfer to occur, the two performances should be as similar as possible). However, others believe that motor abilities are specific [29,30]; that is, the relative performance of an individual on one motor test should provide little or no indication of their relative performance on another test. Our results support positive transfer theory. We found that students’ performance on the Purdue and O’Connor tests improved significantly after phantom training (T1) under direct and indirect vision conditions. The finding that the scores of the dentists on the Purdue and O’Connor tests under direct vision did not improve after one week (unlike students, who showed performance improvements after eight months of training in the phantom course) supports this conclusion (S3 and S4 Appendices). Although significant differences were found after one week of training in the dentist group for the O’Connor task under indirect vision, the improvements of the students at T1 were 4.5 times higher than those of the dentists. Thus, the significant improvement of the students is primarily attributable to phantom training and not to memory effects, which likely explain the improvements of the dentists. Our findings corroborate those of a study showing significant improvements in the performance of dental students due to manual skill training compared with medical students, who showed no improvement [14]. Therefore, training using simple, accessible tools such as the Purdue and O’Connor tasks under indirect vision conditions can significantly improve these abilities. Our results are compatible with studies from the field of occupational therapy that show that practice improves performance [31,32].

Performances on the Purdue and O’Connor tests were preserved when tested at T2. These dexterity tests might not be sufficiently sensitive to detect additional improvement in manual skills after achieving basic capabilities during the intensive phantom course or it is possible that the students have reached their highest performance level. Nevertheless, it is important to identify weak students who have already been admitted to dentistry schools early in their studies to provide them with more training or practice to increase their chance of future success.

Although PD-BH had the highest correlations with final performances, it is one task from a sequence of eight Purdue tasks. However, as noted, all Purdue tests last a very short time and do not simulate the extended concentration required from a dentist, as opposed to the O’Connor test, especially in the novel indirect vision. Moreover, the higher value CV of the O’Connor test under indirect vision indicates the greater differentiation between students.

The test-retest reliability one week apart of the dentists show high correlations (ranged between 0.831 and 0.980) indicating on the internal consistency of these dexterity tests. On the other hand, the students repeated these tests after 8 months and cannot be considered as test-retest because of the major confounding factor, the intensive practicing in the phantom course. Thus, reduction in the correlations is expected because of the significant improvements in the dexterity tests.

There are some limitations of the study. Although significant correlations were found between manual dexterity tests and the final grades in the phantom course and this was repeated in two consecutive years, this study was conducted in the same dental school. In our school, the number of students is small. Further studies in other dental faculties or schools, especially with different admission requirements are needed before conclusive decision can be made regarding the usage of these manual dexterity tests with different vision directions as predictors of potential success in pre-clinical studies.

## Conclusions

1. The O’Connor test under indirect vision is the most appropriate way to monitor and predict the manual skills required of dental students.
2. Three parameters (the initial phantom course grade, the O’Connor test under indirect vision, and the Purdue test using both hands) predict the success of dental students during the initial phases of phantom training.
3. One of the difficulties that students are required to manage in the pre-clinical year is the indirect vision skill. This skill is a learned skill that improves significantly with training. Weak students who experience frustration in their initial attempt to use indirect vision can benefit from exercises in manual dexterity tests from occupational therapy at the beginning of the phantom course.

## Supporting information

S1 Appendix (DOCX)

S2 Appendix (DOCX)

S3 Appendix (DOCX)

S4 Appendix (DOCX)

S5 Appendix (DOCX)
