# Supplementary material for: Predicting the clinical performance of dental students with a manual dexterity test
Source: PLoS One. 2018 Mar 8;13(3):e0193980. doi: 10.1371/journal.pone.0193980 (PMC5843268; doi:10.1371/journal.pone.0193980)
Supplement: S1 Appendix — (DOCX) [file pone.0193980.s001.docx]

S1 Appendix. The correlations analyses between the two manual dexterity tests and between tasks at T0 and T1 for both cohorts of students

| Motor Task | PD-DH | PD-NDH | PD-BH | PD-A | PIND-DH | PIND-NDH | PIND-BH | PIND-A | O-D | O-IND |
| --- | --- | --- | --- | --- | --- | --- | --- | --- | --- | --- |
| PD-DH | 0.422** | 0.492** | 0.366** | 0.275* | 0.475** | 0.234 | 0.452** | 0.338** | -0.128 | -0.210 |
| PD-NDH | 0.470** | 0.562** | 0.657** | 0.489** | 0.525** | 0.448** | 0.469** | 0.498** | -0.360** | -0.251* |
| PD-BH | 0.405** | 0.720** | 0.298* | 0.452** | 0.459** | 0.396** | 0.546** | 0.384** | -0.300* | -0.338** |
| PD-A | 0.377** | 0.421** | 0.403** | 0.516** | 0.335** | 0.387** | 0.342** | 0.506** | -0.279* | -0.287* |
| PIND-DH | 0.352** | 0.524** | 0.505** | 0.180 | 0.319* | 0.422** | 0.487** | 0.374** | -0.228 | -0.421** |
| PIND-NDH | 0.370** | 0.634** | 0.538** | 0.404** | 0.486** | 0.413** | 0.514** | 0.423** | -0.323** | -0.401** |
| PIND-BH | 0.332* | 0.588** | 0.537** | 0.426** | 0.529** | 0.720** | 0.512** | 0.620** | -0.311* | -0.401** |
| PIND-A | 0.316* | 0.412** | 0.312* | 0.638** | 0.290** | 0.529** | 0.606** | 0.370** | -0.356** | -0.284* |
| O-D | -0.101 | -0.455** | -0.368** | -0.367* | -0.133 | -0.359* | -0.208 | -0.150 | 0.703** | 0.351** |
| O-IND | -0.04 | -0.304* | -0.199 | -0.181 | -0.216 | -0.367** | -0.347* | -0.328* | 0.552** | 0.362** |

All correlations are Pearson's correlations. *p<0.05, **p<0.01

Note:

Red – above the diagonal: Correlations between the two manual dexterity tests and between tasks at TO for both cohorts (N=65).

Blue – diagonal: Correlations between the same tasks at T0 and T1 for both cohorts (N=65).

Black – below the diagonal: Correlations between the two manual dexterity tests and between tasks at T1 for both cohorts (N=65).
