# Supplementary material for: Predicting the clinical performance of dental students with a manual dexterity test
Source: PLoS One. 2018 Mar 8;13(3):e0193980. doi: 10.1371/journal.pone.0193980 (PMC5843268; doi:10.1371/journal.pone.0193980)
Supplement: S2 Appendix — (DOCX) [file pone.0193980.s002.docx]

S2 Appendix. Test-Retest Reliability between the Same Tasks of Purdue (N=8) and O'Connor (N=8) at 1^st^ Trial and 2^nd^ Trial for Dentists.

| O-IND | O-D | PIND-A | PIND-BH | PIND-NDH | PIND-DH | PD-A | PD-BH | PD-NDH | PD-DH | Motoric Tasks |
| --- | --- | --- | --- | --- | --- | --- | --- | --- | --- | --- |
|  |  |  |  |  |  |  |  |  | **0.956 | PD-DH |
|  |  |  |  |  |  |  |  | **0.893 |  | PD-NDH |
|  |  |  |  |  |  |  | **0.886 |  |  | PD-BH |
|  |  |  |  |  |  | **0.964 |  |  |  | PD-A |
|  |  |  |  |  | **0.964 |  |  |  |  | PIND-DH |
|  |  |  |  | **0.831 |  |  |  |  |  | PIND-NDH |
|  |  |  | **0.954 |  |  |  |  |  |  | PIND-BH |
|  |  | **0.917 |  |  |  |  |  |  |  | PIND-A |
|  | **0.854 |  |  |  |  |  |  |  |  | O-D |
| **0.980 |  |  |  |  |  |  |  |  |  | O-IN |
