# Supplementary material for: Predicting the clinical performance of dental students with a manual dexterity test
Source: PLoS One. 2018 Mar 8;13(3):e0193980. doi: 10.1371/journal.pone.0193980 (PMC5843268; doi:10.1371/journal.pone.0193980)
Supplement: S3 Appendix — (DOCX) [file pone.0193980.s003.docx]

S3 Appendix. The Mean and Standard Deviation of Purdue and O’Connor Tests of Cohort 1 Students at Three Time Periods and Dentists at 1^st^ Trial*.

| Dentists | Students  (Cohort 1, N=39) | | | Motor Task |
| --- | --- | --- | --- | --- |
| 1^st^ Trial | T2 | T1 | T0 |  |
| Mean  (SD) | Mean  (SD) | Mean  (SD) | Mean  (SD) |  |
| 15.87  (2.32) | 18.00  (1.76) | 17.84  (1.87) | 16.51  (2.01) | PD-DH |
| 14.93  (2.22) | 16.19  1.60)) | 16.31  (1.93) | 14.61  (2.37) | PD-NDH |
| 12.80  (1.82) | 14.15  (1.43) | 13.56  (1.67) | 12.64  (1.59) | PD-BH |
| 38.26  (8.07) | 43.88  (4.77) | 44.10  (6.10) | 39.64  (5.86) | PD-A |
| 10.46  (2.50) | 13.88  (2.38) | 13.18  (1.72) | 11.46  (1.76) | PIND-DH |
| 9.33  (1.91) | 12.12  (1.59) | 11.77  1.84)) | 10.07  (1.81) | PIND-NDH |
| 6.67  (2.61) | 9.68  (1.43) | 8.79  (1.60) | 7.00  (1.69) | PIND-BH |
| 23.06  (5.65) | 33.96  (5.11) | 34.38  5.74)) | 28.23  (5.85) | PIND-A |
| 4.84  (0.49) | 4.68  (0.50) | 4.95  (0.73) | 6.02  (1.26) | O-D |
| 16.67  (4.43) | 12.19  (4.90) | 12.39  (3.19) | 20.14  (6.17) | O-IND |

*Purdue scores are reported in number of pins/parts; O’Connor scores are reported in minutes.
