# Supplementary material for: Predicting the clinical performance of dental students with a manual dexterity test
Source: PLoS One. 2018 Mar 8;13(3):e0193980. doi: 10.1371/journal.pone.0193980 (PMC5843268; doi:10.1371/journal.pone.0193980)
Supplement: S4 Appendix — (DOCX) [file pone.0193980.s004.docx]

S4 Appendix. The Mean and Standard Deviation of Purdue (N=8) and O’Connor (N=8) Tests of the Dentists at 1^st^ trial T0 and a Week Later (Test-Retest, 2^nd^ Trial)*.

| Dentists (N=8) | | Motor Task |
| --- | --- | --- |
| 2^nd^ Trial | 1^st^ Trial |  |
| Mean  (SD) | Mean  (SD) |  |
| 16.62  (3.02) | 16.37  (2.92) | PD-DH |
| 15.37  (2.44) | 15.62  (2.55) | PD-NDH |
| 13.12  (2.10) | 12.37  (1.50) | PD-BH |
| 38.00  (8.96) | 39.00  (9.25) | PD-A |
| 10.5  (2.61) | 10.62  (2.77) | PIND-DH |
| 9.62  (2.13) | 9.12  (1.64) | PIND-NDH |
| 6.87  (2.03) | 6.12  (2.29) | PIND-BH |
| 25.12  (6.64) | 23.37  (7.30) | PIND-A |
| 4.57  (0.39) | 4.81  (0.57) | O-D |
| 16.78  (5.90) | 18.41  (4.97) | O-IND |

*Purdue scores are reported in number of pins/parts; O’Connor scores are reported in minutes.
