# Supplementary material for: Predicting the clinical performance of dental students with a manual dexterity test
Source: PLoS One. 2018 Mar 8;13(3):e0193980. doi: 10.1371/journal.pone.0193980 (PMC5843268; doi:10.1371/journal.pone.0193980)
Supplement: S5 Appendix — (DOCX) [file pone.0193980.s005.docx]

Appendix E. The Mean and Standard Deviation of Purdue and O’Connor Tests of Cohort 2 (N=26) Students at Two Time Periods*.

| Students  (Cohort 2, N=26) | | Motor Task |
| --- | --- | --- |
| T1 | T0 |  |
| Mean  (SD) | Mean  (SD) |  |
| 17.92  (2.01) | 16.80  (2.07) | PD-DH |
| 16.85  (1.70) | 14.84  (1.91) | PD-NDH |
| 14.35  (1.90) | 12.38  (1.69) | PD-BH |
| 42.85  (4.65) | 38.76  (5.42) | PD-A |
| 13.50  (2.17) | 11.11  (1.60) | PIND-DH |
| 11.71  (2.46) | 9.76  (1.68) | PIND-NDH |
| 9.07  (1.59) | 6.96  (1.42) | PIND-BH |
| 30.78  (4.09) | 29.23  (4.07) | PIND-A |
| 5.15  (0.90) | 6.27  (1.14) | O-D |
| 15.30  (4.90) | 26.78  (8.64) | O-IND |

*Purdue scores are reported in number of pins/parts; O’Connor scores are reported in minutes.
